# Assessment of compliance with tetracycline eye ointment treatment to accelerate the elimination of trachoma in Yobe State, Nigeria

**DOI:** 10.1093/inthealth/ihaf014

**Published:** 2025-03-13

**Authors:** Juliana A Amanyi-Enegela, Rinpan Ishaya, Joseph Kumbur, Girija Sankar, William Enan Adamani, Christopher Ogoshi, Nicholas Olobio, Muhammad Babar Qureshi, Caleb Mpyet

**Affiliations:** Inclusive Eye Health and Neglected Tropical Diseases Initiative, CBM Christoffel-Blindenmission Christian Blind Mission e.V, CB1 1BH Cambridge, UK; HANDS, 5A Naomi Jugu Drive, 930103 Rayfield, Jos, Plateau State, Nigeria; CBM Christoffel-Blindenmission, Christian Blind Mission e.V Nigeria Country Office, Abuja, Nigeria; Inclusive Eye Health and Neglected Tropical Diseases Initiative, CBM Christoffel-Blindenmission Christian Blind Mission e.V, CB1 1BH Cambridge, UK; CBM Christoffel-Blindenmission, Christian Blind Mission e.V Nigeria Country Office, Abuja, Nigeria; HANDS, 5A Naomi Jugu Drive, 930103 Rayfield, Jos, Plateau State, Nigeria; Neglected Tropical Diseases Unit, Federal Ministry of Health, PMB 083 Abuja, Nigeria; Inclusive Eye Health and Neglected Tropical Diseases Initiative, CBM Christoffel-Blindenmission Christian Blind Mission e.V, CB1 1BH Cambridge, UK; Department of Ophthalmology, 930003, University of Jos, Plateau State, Nigeria

**Keywords:** elimination, neglected tropical diseases, Nigeria, tetracycline eye ointment (TEO), trachoma, treatment compliance, Yobe State

## Abstract

**Background:**

Trachoma, a neglected tropical disease, remains a significant public health concern in many regions, particularly in sub-Saharan Africa and in Yobe State, Nigeria. One approach for elimination involves administering tetracycline eye ointment (TEO) to children <6 months of age as part of annual mass drug administration (MDA), aligning with the World Health Organization's ‘A’ component of the SAFE (Surgery, Antibiotics, Facial hygiene and Environmental sanitation) strategy for elimination of trachoma as a public health problem. However, suboptimal compliance rates in affected populations pose challenges, potentially serving as a reservoir for reinfection and hindering progress toward trachoma elimination. This study focuses on assessing compliance with topical TEO during MDA and explores strategies to enhance adherence in trachoma-endemic areas of Yobe State, Nigeria.

**Methods:**

A mixed research approach was carried out involving interviews with households across 30 communities in five local government areas where TEO was administered during the 2022 round of MDA. Focus group discussions were conducted with subsets of the population who received TEO to gain insights into the underlying reasons for non-compliance and ways to improve compliance. Additionally, healthcare provider perspectives on treatment administration, compliance and community health education were explored.

**Results:**

Findings from this study show that there is already a high level of compliance with TEO usage, however, forgetfulness due to competing domestic chores, insufficient quantity of TEO given for 6-week applications, low awareness about the impact of trachoma infection on the eyes and the stinging feeling after application, especially in children <6 months of age, are some barriers that affect TEO usage compliance. Following up with TEO recipients would remind them to use the ointment as required, while incentivizing health workers and community drug distributors to conduct follow-up visits to households, increasing awareness on the impact of trachoma on the eyes. Increasing the quantity of TEO allocation would also improve compliance.

**Conclusions:**

Yobe State has made remarkable progress towards eliminating trachoma as a public health problem, as 12 local government areas no longer require MDA. Sustaining this momentum means ensuring high compliance among the population eligible to receive TEO to prevent any reservoir for reinfection in the state.

## Introduction

Trachoma, a neglected tropical disease caused by repeated infections of the bacterium *Chlamydia trachomatis*, is a leading cause of preventable blindness worldwide.^1^ It primarily affects populations living in impoverished areas with limited access to healthcare and proper sanitation facilities.^2^ Despite global elimination efforts, trachoma is still a public health problem in 42 countries across Africa, Central and South America, Asia, Australia and the Middle East, where poverty, overcrowding, limited access to and use of water and poor sanitation and hygiene practices enable it to thrive.^3^

The World Health Organization (WHO)^4^ recommends the Surgery, Antibiotic, Facial hygiene and Environmental improvement (SAFE) strategy for eliminating trachoma as a public health concern in endemic regions. The ‘A’ component of the SAFE strategy entails mass drug administration (MDA), a pivotal element in trachoma elimination initiatives proven to effectively curb disease transmission and progression. MDA involves annually administering a broad-spectrum antibiotic, typically azithromycin tablets and oral suspension, to the eligible at-risk population through directly observed treatment.

Tetracycline eye ointment (TEO) is commonly administered during MDA campaigns, at least twice daily for 6 weeks, primarily for infants <6 months of age and individuals ineligible for azithromycin tablets and suspension. While the ingestion of azithromycin tablets and oral suspension is directly observed, the application of TEO by recipients is unsupervised. These individuals are expected to apply TEO topically for the recommended duration.

The success of trachoma elimination hinges on achieving high treatment coverage and compliance rates. A minimum of 80% coverage, coupled with surgery and facial hygiene and environmental improvement (F&E) activities, has demonstrated effectiveness in reducing trachoma transmission, curbing its spread and preventing long-term complications.^3,5^

Nigeria is one of 42 countries where trachoma remains a public health problem and Yobe State is one of the four states with a prevalence of trachomatous inflammation–follicular (TF) ≥5% in children 1–9 y of age and trachomatous trichiasis (TT) ≥0.2% in people ≥15 y of age.^6^ While 12 out 17 endemic local government areas (LGAs) have stopped MDA, trachoma continues to pose a considerable burden on affected communities, as 5 LGAs still require at least one round of annual MDA in addition to S, F&E activities.

This research aimed to evaluate TEO treatment compliance, understanding the barriers and facilitators to adherence, and explored ways of improving TEO usage compliance in trachoma-endemic LGAs in Yobe State, Nigeria.

## Methods

### Study design

The study was conducted about 7 weeks after the trachoma MDA was completed in Yobe State. This assessment utilized a multimethod cross-sectional design comprising household interviews, in-depth interviews and focus group discussions (FGDs). This descriptive multimethod design focused on a mixed research approach. Interviews and data collection were between June and the end of July 2022.

#### Quantitative and qualitative methods

The quantitative method in this study was the key informant interviews conducted across 152 households while the qualitative methods were in-depth interviews (IDIs) and FGDs. A total of 36 in-depth interviews were conducted with health workers while five FGDs were also conducted (one FGD in each LGA). Experienced research data collectors used a structured interview questionnaire for the household interviews, a semi-structured guide was used for the IDI and an FGD guide was used to facilitate the FGDs.

### Study sites

**Figure 1.**
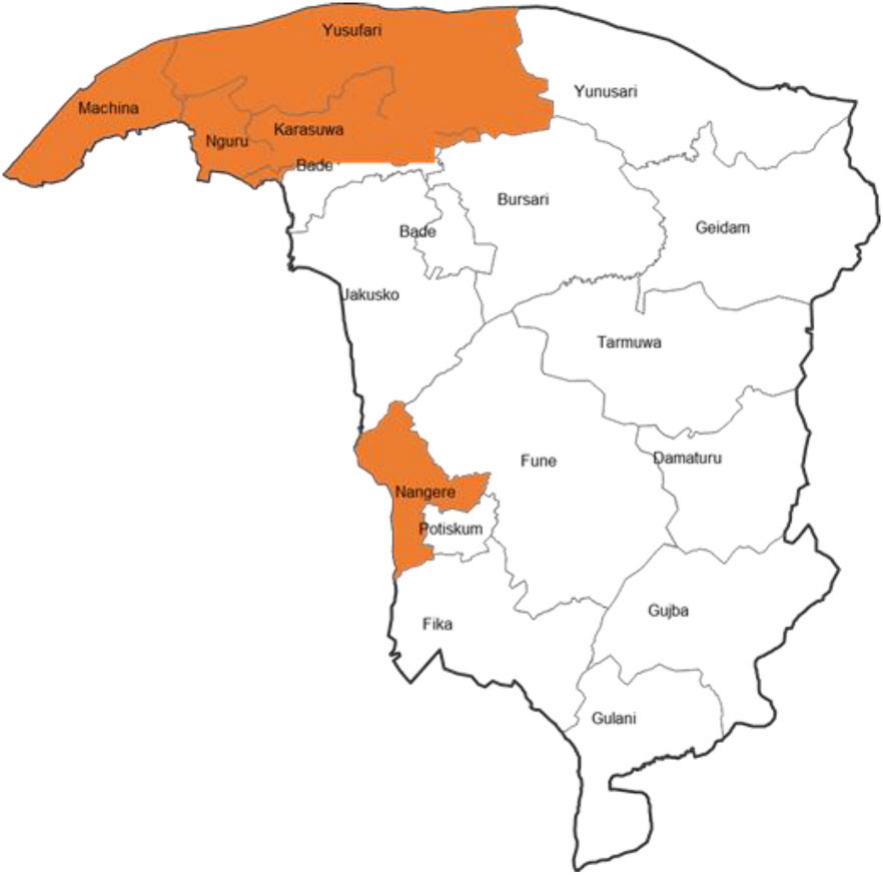
Map of Yobe State showing the five study sites.

The assessment was carried out in five LGAs (Karasuwa, Machina, Nengere, Nguru and Yusufari), as shown in Figure 1.

Karasuwa, Machina and Nguru LGAs are bordered by Jigawa State, while Nengere and Yusufari LGAs are bordered by Bauchi State and the Republic of Niger, respectively.

### Study participants and data collection procedure

A hybrid of purposive and random sampling methods was employed to designate LGAs, wards and communities for the study. The selection of the five LGAs was purposive, guided by previous impact assessments showing that these areas have not yet met the WHO-recommended threshold for discontinuing MDA, despite completing the required rounds based on baseline prevalence. This highlights the need to explore the factors contributing to active and persistent trachoma cases in these areas. Investigating these underlying causes will enable targeted interventions, helping to address ongoing transmission and achieve the WHO standards for elimination.

From each LGA, two wards were selected randomly, and within each selected ward, three communities were randomly chosen to participate in the research.

At the community level, the Community Drug Distributors (CDDs) register served as the primary sampling unit. Community members who had received TEO during the recent MDA were randomly selected to participate in the assessment. One FGD was conducted per LGA and one frontline health worker was interviewed in each of the selected communities.

## Ethics statement

Approval for this study was obtained from the Yobe State Health Research Ethics Committee. Prior to commencing fieldwork, all communities within the study areas were briefed on the research objectives through their traditional leaders. The research team provided a detailed explanation of the study's goals to interviewees and members of the FGDs. No personal identifiers were employed and all interviewers underwent training in research ethics and the sensitive conduct of interviews. Furthermore, both written and verbal consents were obtained and participants were assured of the confidentiality of their information. The total number of interviews conducted is shown in Table 1.

**Table 1:** Data collection table.

LGAs/research activities	Karasuwa	Machina	Nengere	Nguru	Yusufari	Total
IDIs with community members	31	31	30	30	30	152
Health worker interviews	8	7	8	8	5	36
FGDs	1	1	1	1	1	5

## Data management and analysis

Information obtained from household interviews and IDIs was documented without any personal identifiers and securely stored in password-protected computers. The FGDs were conducted in the local language and recorded using an end-to-end encrypted device. Subsequently, the recorded FGDs underwent transcription and translation into English for analysis.

Excel (Microsoft, Redmond, WA, USA) and NVivo 12 (Lumivero, Denver, CO, USA) software were used to organize the qualitative data categories for analysis. After data familiarization, the data were coded manually and the transcripts were transferred to NVivo 12 to organize the data and increase the thoroughness of the coding process. Thematic analysis was used to explore patterns and themes within the data.

## Results

### Demography of respondents

#### Sociodemographics and background characteristics

The survey was conducted in 30 communities spanning 10 wards across five LGAs—Karasuwa, Machina, Nengere, Nguru and Yusufari—where trachoma MDA took place in Yobe State in 2022. A total of 188 individuals, including health workers, participated in the interviews, with representation distributed at 20% per LGA. The majority of respondents were women <40 y of age, constituting 57% of the surveyed population. Further details on the demographic composition of respondents can be found in Table 2.

**Table 2.** Sociodemographics of interview respondents.

Description		Interviewees, n (%)
Demographic features of respondents		Number of respondents (households, 152; health workers, 36; total respondents, 188)
Age group (years)	15–34	107 (57)
	35–54	52 (28)
	55–74	21 (11)
	≥75	8 (4)
Gender	Female	145 (77)
	Male	43 (23)
Occupation	Housewife	75 (40)
	Health worker	36 (19)
	Farmer	33 (18)
	Trader	31 (16)
	Civil servant	13 (7)
Responding on behalf of someone (parent/caregiver)	Yes	43 (23)
	No	145 (77)

### Quantitative findings

#### TEO compliance during trachoma MDA

**Figure 2:**
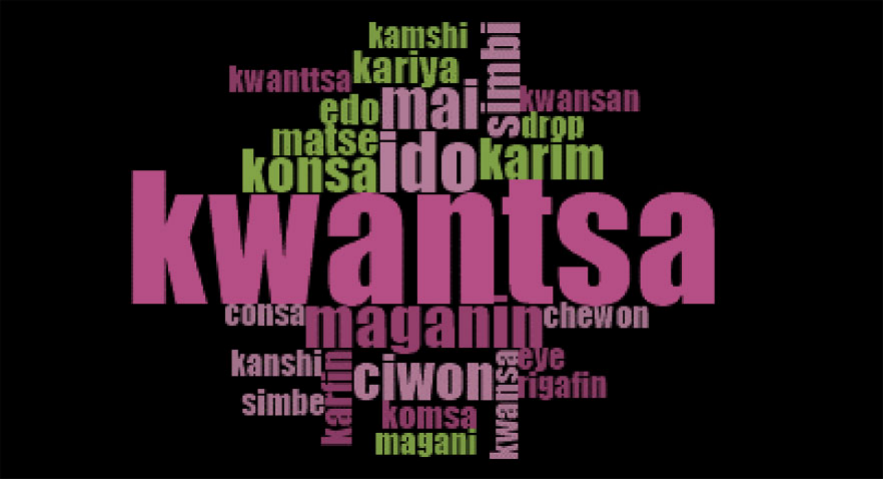
Word cloud of local names for TEO in Yobe State.

A total of 151 (99%) respondents acknowledged receiving TEO during the 2022 trachoma MDA. Of these, 78 (52%) affirmed knowledge of the specific name of the eye ointment received during MDA, while 127 (84%) confirmed awareness of a local name for TEO. The details are presented in Table 3 and Figure 2.

**Table 3.** Knowledge of respondents on TEO

Description	Response, n (%)	
	Yes	No
At least one person received TEO in the household during the 2022 trachoma MDA	151 (99)	1 (1)
Know the name of the ointment given during MDA	78 (52)	73 (48)
Know a local name for the eye ointment given during trachoma MDA	127 (84)	24 (16)

**Figure 3.**
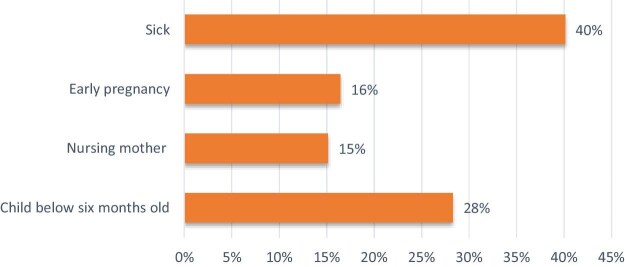
Reason for receiving TEO during MDA.

Regarding the rationale for receiving TEO during trachoma MDA, 40% of respondents affirmed that illness prompted their receipt of TEO. Additionally, 28% indicated that TEO was administered for children <6 months of age, while 16% and 15% mentioned an early stage of pregnancy and new nursing mothers (>2 weeks postpartum), respectively, as the basis for receiving TEO during MDA. This is illustrated in Figure 3.

## Respondent's understanding of TEO usage

Of the total respondents, 91% (n=138) affirmed that they were using TEO for the first time during the trachoma MDA. Meanwhile, 7% reported having received TEO twice and 2% indicated three instances of receiving TEO during the annual MDA. Almost the entire respondent pool, 99%, mentioned that community volunteers explained how to use TEO and they understood the provided guidance. Regarding the application process, 92% found it easy while 8% experienced difficulty.

**Figure 4.**
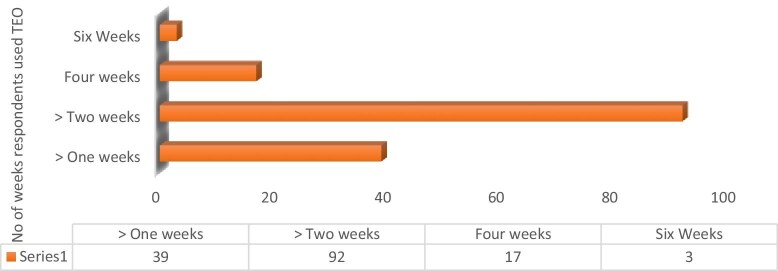
Respondent's duration of TEO usage.

In terms of the quantity of TEO received during the 2022 trachoma MDA, 64% (n=97) received two tubes, 34% (n=51) received one tube and 2% (n=3) received three tubes. The frequency of TEO application varied, with 58% applying it twice daily (morning and evening), 31% applying it three times a day and 11% applying it once daily. Table 4 summarizes the aforementioned details and Figure 4 provides an overview of the duration of use based on household responses.

**Table 4.** Respondent's understanding of TEO usage

Description	Response, n (%)	
	Yes	No
Respondent's first time of using the TEO	138 (91)	13 (9)
The community volunteer explained when and how to apply the TEO	150 (99)	1 (1)
Respondent understood the instructions on how to use the TEO?	150 (99)	1 (1)
It was difficult to apply on the everted eyelid	11 (8)	140 (92)
Number of times respondent has received TEO during annual MDA	1	138 (91)
	2	12 (8)
	3	2 (1)
Number of TEO tubes respondent received during 2022 MDA	1	51 (34)
	2	97 (64)
	>2	3 (2)
Was the quantity of TEO given sufficient for the recommended duration of usage?	114 (76)	1 (24
Did you miss applying TEO within the period it was used?	20 (13)	132 (87)
Did you use ointment for other purposes?	1 (1)	151 (99)
Community volunteer or health worker visit to household to follow-up on the TEO usage?	119 (78)	33 (22)

**Figure 5.**
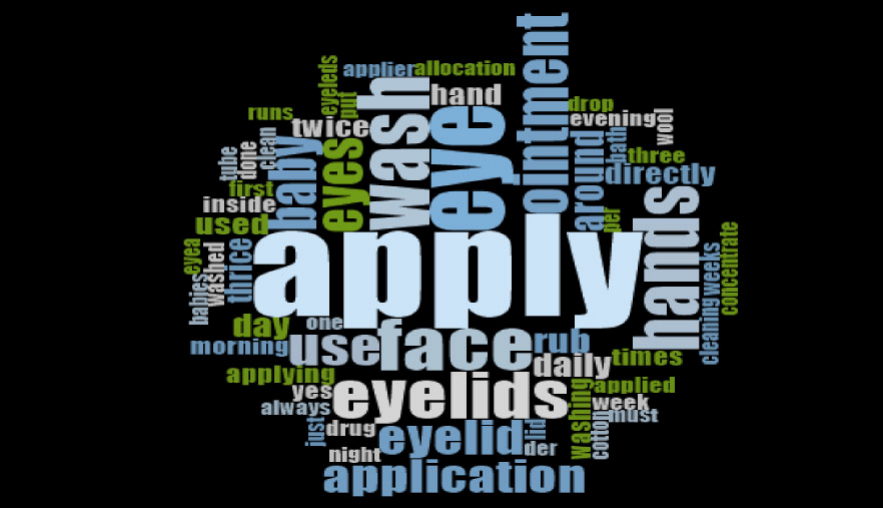
Word cloud showing participant's responses on how to use TEO.

All the respondents were able to provide a brief explanation of how to use TEO. Respondents indicated that their understanding was based on the explanation provided by the community volunteer during MDA. A large proportion of the respondents also mentioned hand hygiene (washing of hands) before applying TEO to the eyelids. Figure 5 is a word cloud showing the words used by respondents while explaining TEO usage for prevention or treatment of trachoma.

### Qualitative findings

Concerning the familiarity and practical application of TEO, a significant majority of respondents expressed positive experiences. Many reported relief from itchy eye conditions, attributing the positive outcomes to the use of TEO. Additionally, some participants highlighted that their trust in community volunteers and health workers played a crucial role in motivating them to adhere to the prescribed treatment. The overall satisfaction with the effectiveness of the medicine was unanimous among respondents, leading to requests for additional supplies. Below are some of the responses:

I applied the TEO on my eyelids mornings and evenings. Before now my eyes were itching and red due to continuous itching. But after applying the medication, there was a stinging feeling, and my eyes were teary. I was completely relieved after one week of using TEO I really enjoyed it. I will suggest that more than two tubes is provided as two tubes was not sufficient for me to use for two weeks. — NEN_RA55

 

I received the TEO ointment on behalf of my 3-month-old child. We were told that it will prevent eye disease (trachoma), so I used it as directed because I do not want my innocent child to go blind. I applied it in the morning and evening after bathing my daughter. I received two tubes and used it for two weeks. We are grateful to the suppliers of this medicine. — KAR_RA44

Several factors contributing to non-usage and non-compliance during the recommended period were identified by respondents. Some acknowledged missing TEO applications due to household responsibilities, forgetfulness, busy farming schedules, fear of using expired medication and, in certain cases, low awareness. However, the involvement of CDDs and health workers in follow-up efforts played a pivotal role in improving TEO usage and adherence. Women receiving TEO for infants <6 months of age expressed challenges in applying the medication to the eyelids. Moreover, a prevalent concern among participants was that the quantity of provided TEO was insufficient for the recommended duration of usage.

Below are samples of comments by participants:

There was total compliance in my ward because of its importance, however, some people may not be keen to use TEO due to poor awareness about the benefits of using the ointment. — MACH_HW22

 

I think sometimes we get busy with house chores and farm work, and this makes some persons forget to use TEO as recommended. Sometimes it was difficult to apply it on the eyelid of my small child and I was afraid that the stinging feeling will affect my child. However, the health worker assured me that there would be no harm if I use it correctly, I trust their judgement, so I used it as advised. I know I am protecting my daughter's eyes. — NGU_RA33

 

For me, I was not sure about the expiration date and I was afraid of the side effect, but the CDD encouraged me to use the TEO for my child as the benefits are enormous. I also think these two factors that can be responsible for non-compliance. — YUS_HW11

 

Poor follow-up could result in low usage because people may forget; the nature of our work is farming, we leave home early and come back late sometimes, this makes it difficult to remember to use TEO as advised. — NEN_HW55

In addressing ways to enhance compliance among the population receiving TEO during trachoma MDA, respondents (health workers) affirmed the existing high compliance level but suggested additional measures. They emphasized the importance of intensifying awareness campaigns regarding the significance of TEO usage. Furthermore, they proposed incentivizing CDDs or collaborating with community town announcers to deliver daily reminders to community members. The following are excerpts from responses provided by both community members and health workers:

Our community volunteers are hardworking, maybe community announcers can remind people to use the eye ointment every day because people may forget to use it due to other priorities. The duration of application is long, people may get tired, however, the suggested CDD and health workers visits to households who received TEO during MDA will encourage them to use it, thus improving compliance. — KAR_HW33

 

It is important for people to know why they are being given the TEO in the first place. Some may forget, but creating more awareness on the benefits of TEO, especially its role in preventing blinding infection in target communities would definitely improve compliance. — NGU_HW44

 

Awareness and engagement of more CDDs would increase TEO usage. Additional incentives should also be provided for CDDs and health workers to encourage follow up in the communities. — NEN_HW44

**Figure 6.**
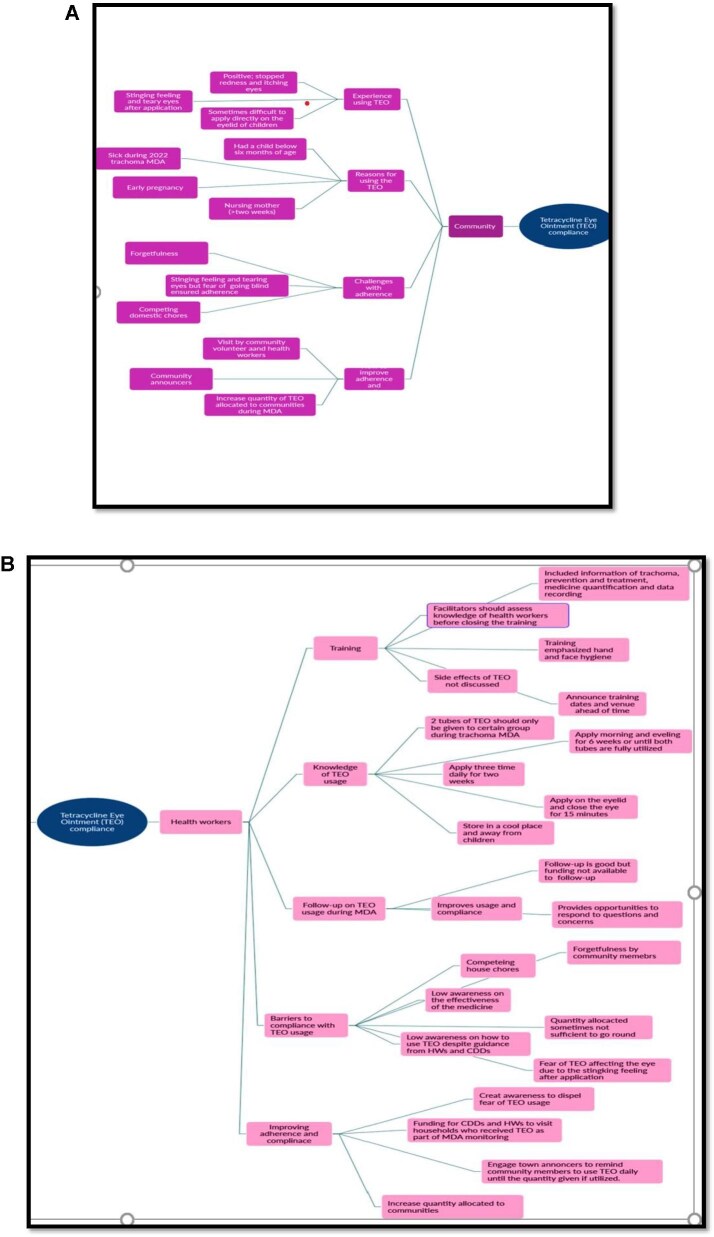
Mind map of responses on TEO compliance provided by **(A)** community members and **(B)** health workers.

Figure 6 is a mind map showing the responses provided by community members and health workers on the facilitators and barriers of utilization of TEO and compliance during MDA.

## Discussion

The study aimed to assess the adherence level for the application of topical TEO and to investigate methods for enhancing compliance with the TEO regimen in trachoma-endemic districts within Yobe State, Nigeria. Compliance, in a broad sense, entails adhering to a rule, guideline or specified course of action. In the context of this research, compliance specifically denotes the consistent application of the TEO received during MDA, ensuring it is used at least once daily for 6 weeks or until the provided TEO is depleted.

The study's findings revealed that 99% (n=151) of the interviewed households received TEO for specific purposes. The distribution included households with children <6 months of age, women in early pregnancy, new nursing mothers (<2 weeks) and individuals with illnesses within the community. Although this aligns with the WHO’s recommendations^7^ for eligible TEO recipients, it is noteworthy that pregnancy and breastfeeding are not contraindications for azithromycin.

Across the 10 wards visited in the five LGAs, 100% compliance was reported in 9 wards, with only 1 reporting 99%. Respondents widely acknowledged the usefulness of guidance provided by community volunteers during MDA, with 99% being able to recall the steps required for safe TEO application. Most participants exhibited awareness of trachoma, cited at least one local name for TEO and emphasized the importance of hand and face hygiene before applying TEO to the lower eyelids—consistent with health workers’ pre-MDA training and findings in Ethiopia,^8^^–^^1^^0^ which underscored the significance of general trachoma knowledge and prior MDA information in participation and coverage.

While feedback varied on daily application frequency and duration, the WHO^7^ recommends applying TEO twice daily for 6 weeks. Although only 2% confirmed using TEO for the recommended period, the majority (98%) used it between 1 and 4 weeks until depletion, prompting a call for increased TEO tube allocation during MDA.

The study findings showed that a factor contributing to compliance was effective guidance from community volunteers and health workers during trachoma MDA. It was evident that community members possessed the necessary information on TEO usage, underscoring the crucial role of CDDs and the quality of pre-MDA training provided. However, inadequate training for health workers and CDDs, as indicated by Krentel et al.,^11^ can negatively impact MDA compliance, emphasizing the importance of maximizing the performance of CDDs and health workers in achieving NTD elimination goals.

Trust and confidence in the CDDs emerged as key factors influencing community members’ comfort in using TEO. The fact that CDDs and health workers are integral members of the community and participate in other public health interventions underscores the essential role of trust in shaping individual acceptance of MDA, a point highlighted in Krentel et al.’s review.^11^ The perceived benefits of TEO also played a crucial role in shaping community attitudes towards compliance as the fear of visual impairment served as a significant motivator for compliance among caregivers of recipients >6 months of age. This is consistent with the findings by Mitchell et al.^12^ and Bontha and Suchismita^13^ in Fiji and India, respectively, where knowledge of the potential health benefit of scabies and lymphatic filariasis MDA facilitated acceptance and compliance. This highlights the benefits of pre-MDA community mobilization and sensitization regarding the risks of preventive chemotherapy in NTDs.

Follow-ups by CDDs and health workers were an important support system that served as reminders for community members to use TEO in certain instances. Notably, the decrease in the prevalence of TF in these LGAs over time may be partly attributed to the high compliance with MDA.

Although there was reported high compliance, the application of TEO stands out as a component of trachoma MDA that is not directly observed, unlike the ingestion of azithromycin and oral suspension. This lack of direct observation necessitates a commitment to comply without follow-up, making it challenging to verify the accuracy of reported actions. Forgetfulness, often attributed to competing domestic chores, emerged as a commonly cited reason for non-compliance. Additionally, the stinging sensation post-application posed a potential deterrent for caregivers, particularly when applying TEO to children <6 months of age. This aligns with West’s^14^ assertion that compliance with TEO usage tends to be subpar due to the recommended duration for effectiveness and discomfort arising from its oily base and stinging effect on the eyelid.

Community members widely agreed that the quantity of provided TEO was insufficient for continuous use throughout the recommended 6-week application period. Health workers echoed concerns about low awareness regarding the impact of trachoma and fear of expiry dates and side effects as factors contributing to non-compliance. These findings resonate with a trachoma MDA coverage survey in Ethiopia conducted by Tilahun and Fenta^9^ and Feyisa et al.,^10^ where low coverage was linked to inadequate awareness, leading to misconceptions among household members. A similar sentiment was reported in a trachoma coverage evaluation survey in Ethiopia by Bekuma et al., ^15^ where >19% of the population expressed fear of azithromycin's side effects.

## Conclusions

MDA stands as a crucial pillar in the trachoma elimination strategy. Over time, the global landscape of trachoma has evolved, with approximately 15 countries receiving WHO validation for successfully eliminating trachoma as a public health concern.^16^ Recent survey findings indicate the cessation of trachoma MDA in 12 LGAs in Yobe State, while 5 LGAs still have at least one round of MDA. The findings of this study indicate that while compliance levels are commendably high, further efforts are required to maintain and possibly enhance this compliance during trachoma MDA. To address issues of forgetfulness among community members, the study proposes the engagement of additional community volunteers for household visits throughout the treatment period. An alternative strategy could involve utilizing community announcers to provide daily reminders for the application of TEO.

Moreover, the consensus underscores the critical need to heighten awareness regarding the severe visual impairments associated with untreated trachoma. Enhancing understanding of the disease's impact is expected to motivate community members to utilize the provided TEO.

Incentivizing CDDs and health workers could further motivate these key personnel to diligently follow up with populations that have received TEO, ensuring adherence beyond the MDA campaign. Assessing the knowledge of health workers and CDDs post-training is also crucial to confirm their understanding of the correct information to disseminate during the MDA.

Finally, it is imperative to standardize the distribution of TEO to ensure that each eligible individual receives the recommended two tubes. The study highlighted discrepancies in distribution, with some receiving fewer than two tubes and others more. Implementing robust systems for the quantification and allocation of TEO to endemic LGAs will help in aligning with WHO recommendations and ensuring equitable and effective distribution.

## Data Availability

The datasets generated and analyzed during the current study are available from the corresponding author upon reasonable request.

## References

[1] World Health Organization . Trachoma fact sheet. 2020. Available from: https://www.who.int/news-room/fact-sheets/detail/trachoma [accessed 8 March 2021].

[2] Habtamu E, Wondie T, Aweke S et al. Trachoma and relative poverty: a case-control study. PLoS Negl Trop Dis. 2015;9(11):e0004228. 26600211 10.1371/journal.pntd.0004228 PMC4657919

[3] World Health Organization . WHO Alliance for the Global Elimination of Trachoma by 2020: progress report, 2019. Wkly Epidemiol Rec. 2020;95(30):349–60. https://www.who.int/publications/i/item/who-wer9530

[4] World Health Organization Fifty-First World Health Assembly. WHA51.11. Agenda item 20. Global elimination of blinding trachoma. Available from: https://iris.who.int/bitstream/handle/10665/79806/ear11.pdf [accessed 8 March 2021].

[5] Chidambaram JD, Lee DC, Porco TC et al. Mass antibiotics for trachoma and the Allee effect. Lancet Infect Dis. 2005;5(4):194–6. 15792732 10.1016/S1473-3099(05)70032-3

[6] Federal Ministry of Health, Nigeria . National Neglected Tropical Diseases Nigeria Multi-Year Master Plan 2015–2020. Available from: file:///Users/laurabader/Downloads/Neglected%20Tropical%20Diseases%20-%20Nigeria%20Multi-Year%20Master%20Plan%20(2015-2020)_1661784168.pdf [accessed 27 February 2025].

[7] World Health Organization Trachoma control: a guide for programme managers. Available from: https://iris.who.int/bitstream/handle/10665/43405/9241546905_eng.pdf?sequence=1 [accessed 29 June 2021].

[8] Ebert CD, Astale T, Sata E et al. Population coverage and factors associated with participation following a mass drug administration of azithromycin for trachoma elimination in Amhara, Ethiopia. Trop Med Int Health. 2014;24(4):493–501. 10.1111/tmi.13208 PMC6850572 30674087

[9] Tilahun Z, Fenta TG. Coverage of azithromycin mass treatment for trachoma elimination in north-western Ethiopia: a community based cross-sectional study. BMC Ophthalmol. 2018;18:193. 30081851 10.1186/s12886-018-0868-1 PMC6091195

[10] Feyisa T, Bekele D, Tura B et al. To eliminate trachoma: azithromycin mass drug administration coverage and associated factors among adults in Goro district, Southeast Ethiopia. PLoS Negl Trop Dis. 2022;16(6):e0010169. 35759466 10.1371/journal.pntd.0010169 PMC9236244

[11] Krentel A, Fischer PU, Weil GJ. A review of factors that influence individual compliance with mass drug administration for elimination of lymphatic filariasis. PLoS Negl Trop Dis. 2013;7(11):e2447 24278486 10.1371/journal.pntd.0002447 PMC3836848

[12] Mitchell E, Tavui A, Andersson S et al. Acceptability of a nationwide scabies mass drug administration (MDA) program in Fiji: a qualitative interview-based study. Lancet Reg Health West Pac. 2024;51:101194. 39295851 10.1016/j.lanwpc.2024.101194 PMC11408017

[13] Bontha VB, Suchismita M. Mass drug administration under the programme to eliminate lymphatic filariasis in Orissa, India: a mixed-methods study to identify factors associated with compliance and non-compliance. Trans R Soc Trop Med Hyg. 2008;102(12):1207–13. 18632125 10.1016/j.trstmh.2008.05.023

[14] West SK . Azithromycin for control of trachoma. Community Eye Health. 1999;12(32):55–6. 17492006 PMC1706032

[15] Bekuma TT, Mosisa Kebebew G, Desalegn Waktole Z et al. Coverage assessment survey following trachoma mass drug administration (MDA) in six districts of Oromia, Western Ethiopia, 2017. PLoS Negl Trop Dis. 2019;13(12):e0007924. 31841516 10.1371/journal.pntd.0007924 PMC6936871

[16] World Health Organization . Trachoma fact sheet. 2022. Available from: https://www.who.int/news-room/fact-sheets/detail/trachoma [accessed 29 October 2022].

